# The Association of Uric Acid Calculi with Obesity, Prediabetes, Type 2 Diabetes Mellitus, and Hypertension

**DOI:** 10.1155/2017/7523960

**Published:** 2017-08-23

**Authors:** Fang-Yeh Chu, Chih-Chun Chang, Pin-Hao Huang, Yi-Ning Lin, Po-Wen Ku, Jen-Tang Sun, Jung-Li Ho, Tzung-Hai Yen, Ming-Jang Su

**Affiliations:** ^1^Department of Clinical Pathology, Far Eastern Memorial Hospital, New Taipei, Taiwan; ^2^School of Medical Laboratory Science and Biotechnology, Taipei Medical University, Taipei, Taiwan; ^3^Graduate School of Biotechnology and Bioengineering, Yuan Ze University, Taoyuan, Taiwan; ^4^Department of Medical Laboratory Science and Biotechnology, Yuanpei University, Hsinchu, Taiwan; ^5^Division of Urology, Department of Surgery, Far Eastern Memorial Hospital, New Taipei, Taiwan; ^6^Department of Emergency Medicine, Far Eastern Memorial Hospital, New Taipei, Taiwan; ^7^Division of Nephrology and Clinical Toxicology, Chang Gung Memorial Hospital, Linkou Medical Center, Taoyuan, Taiwan

## Abstract

**Objectives:**

To disclose the link between the composition of urolithiasis, especially that of uric acid calculi, and obesity, prediabetes, type 2 diabetes mellitus, and hypertension.

**Materials and Methods:**

Patients who had urinary calculi and underwent surgical treatment were registered in the study. The composition of urinary calculi was analyzed and correlated with clinical features and biomedical profiles of the patients before surgical intervention.

**Results:**

A total of 666 patients with urolithiasis who underwent surgical management were registered and analyzed. In those who had uric acid calculi, there was a significant association with prediabetic (OR: 20.11, 95% CI: 7.40–54.63, *P* < 0.001) and diabetic states (OR: 11.55, 95% CI: 4.41–29.97, *P* < 0.001). It also seemed that uric acid calculi were related to obesity but there was no statistical significance (OR: 2.45, 95% CI: 0.91–6.62, *P* = 0.078). There was no association of uric acid calculi with hypertension (OR: 1.08, 95% CI: 0.54–2.17, *P* = 0.822) and concurrent urinary tract infection (OR: 0.93, 95% CI: 0.44–1.96, *P* = 0.841).

**Conclusion:**

There was a remarkable association of uric acid calculi with prediabetic and diabetic states. The uric acid calculi were also seemingly associated with obesity in patients with urolithiasis undergoing surgical management.

## 1. Introduction

It was noticeable that the prevalence of urolithiasis has been rising globally over the past decades [1, 2], along with the upward trend in prevalence of obesity, impaired glucose tolerance, and diabetic states [3, 4]. Though not fully explained, it was disclosed that diabetic states were remarkably associated with increased risk of urinary stone disease [5, 6]. In addition to diabetes, it was reported that obesity, hyperlipidemia, hyperuricemia, and hypertension (HTN) could be the risk factors of developing urolithiasis [6–10]. Further investigation revealed that type 2 diabetes mellitus (T2DM) and high body mass index (BMI) were particularly attributed to uric acid calculi formation [11–13]. Besides, a recent study indicated that increased levels of calcium, oxalate, and uric acid, known as risk factors for developing urolithiasis, were observed in the urinary excretion of the obese population, while the urinary concentration of citrate, which prevented urinary stone formation, was also increased in the same population [14]. The role of urinary stone composition in these metabolic disorders remains obscure and further investigation is required.

In this study, we aimed to investigate the association of calculi composition, particularly that of uric acid calculi with obesity and prediabetic and diabetic states as well as HTN in patients who underwent surgical management for urolithiasis.

## 2. Materials and Methods

### 2.1. Subjects and Experimental Protocol

This retrospective study was conducted in Far Eastern Memorial Hospital (FEMH) during July 2015 to July 2016. The study was reviewed and approved by the research ethics review committee of FEMH and was conducted in accordance with the Declaration of Helsinki in 1964. All adult Taiwanese patients (more than 20 years old) who had urinary calculi and received surgical treatment were registered in this investigation. The stone or stone fragments were extracted and analyzed for chemical composition. If a patient had repeated examination for chemical composition of urinary calculi, results of the first exam were adopted for data collection and analysis. Besides, the clinical data, including patient age, gender, BMI, preoperative laboratory survey [such as plasma glucose, glycated hemoglobin (HbA1c), serum creatinine, and routine urinalysis], personal history of T2DM and HTN, and medication use for T2DM and HTN, were collected via the electronic chart review. The calculation of BMI depended on the patient's weight and height [BMI = weight (kg)/height (m^2^)]. Obesity was considered if a patient had a BMI of 30 kg/m^2^ or more. The diagnostic criteria of prediabetes and T2DM were complied with the guideline of American Diabetes Association (ADA) [15, 16]. Prediabetes was considered if a patient was found to have impaired fasting glucose (a plasma glucose level more than 100 mg/dL but less than 126 mg/dL) or impaired glucose intolerance (an HbA1c level more than 6.0% but less than 6.5%) with neither oral hypoglycemic agents (OHAs) use nor insulin therapy. T2DM was considered if a patient had a plasma glucose level of 126 mg/dL and more and an HbA1c level of 6.5% and more or underwent medical treatment of OHAs or insulin on admission. HTN was considered if a patient had at least 2 consecutive measures of systolic/diastolic blood pressures, being 140/90 mmHg and more, or had a history of HTN with/without use of antihypertensive agents on admission. Furthermore, urinary tract infection was considered if patient had any infectious symptoms or signs (such as fever, dysuria, increased urinary frequency, and urine incontinence), pyuria and bacteriuria found on urinalysis, or positive result of urine culture collected before operation. The biochemical laboratory profiles were determined by the automated chemistry (Hitachi 911, Roche, MN, USA) or urine analyzers (Clinitek Atlas, Siemens, IN, USA), all according to the manufacturer's protocols. The chemical composition of urinary calculi was analyzed by the Fourier transform infrared spectroscopy (FTIR 4100, Jasco, MD, USA). The calculi composition of 50% and more was considered predominant; otherwise, it was considered as mixed type.

### 2.2. Statistical Analysis

All data were presented as median (interquartile range, IQR) or number (percentage). The Mann–Whitney *U* test was used to compare clinical variables between groups. The association of uric acid calculi with obesity, prediabetic and diabetic states, HTN, and concurrent urinary tract infection was initially assessed using the univariate logistic regression analysis. To control for confounding factors, a multivariate binary logistic regression analysis was further performed. The results were presented as odds ratio (OR) with 95% confidence interval (CI). A *P* value less than 0.05 was considered statistically significant. All statistical analysis was performed using SPSS statistical software (version 19.0; SPSS Inc., Chicago, USA).

## 3. Results

During the study interval, 761 patients underwent surgical management for urinary stone diseases in FEMH and a total of 825 extracted stones/stone fragments were sent for composition analysis. Of these, 95 patients were excluded because of incomplete clinical data, and 666 patients were eventually recruited for analysis. The baseline features of patients who underwent surgical management for urinary stone diseases were described in Table 1. In the study population, the median age was 55 years and the male gender accounted for 69.4% (*n* = 462). Besides, 112 (16.8%) of the participants had obesity (BMI of 30 kg/m^2^ and more) and 348 (52.3%) were overweight (BMI of 24 kg/m^2^ and more but less than 30 kg/m^2^), whereas 206 (30.9%) had a BMI less than 24 kg/m^2^. Among these patients undergoing surgical management for urolithiasis, 69 (10.4%) were found to have prediabetes with impaired fasting glucose and 149 (22.4%) had type 2 diabetes mellitus with dietary control or oral antihyperglycemic agents or insulin therapy, whereas 259 (38.9%) had HTN with or without antihypertensive medication use. Meanwhile, 184 (27.6%) with urinary stone disease were found to have concurrent urinary tract infection before surgical management. The median serum creatinine of this study population was 0.97 mg/dL. Both the median plasma glucose and HbA1c levels were significantly higher in the diabetic and prediabetic group than those in the nondiabetic group (*P* < 0.001). Furthermore, the median systolic and diastolic blood pressures were significantly higher in the hypertensive group than that in the nonhypertensive group. Among the study population, those who had calcium oxalate calculi accounted for 63.5% (*n* = 423) and who had calcium phosphate calculi accounted for 26.1% (*n* = 174), whereas those who had uric acid calculi accounted for 6.9% (*n* = 46). Additionally, 11 had magnesium ammonium phosphate calculi, 3 had protein calculi (including cystine), and still 9 had mixed type of urinary stones.

As listed in Table 2, multivariate logistic regression analysis was performed for realizing the association of urolithiasis composition with HTN, obesity, and prediabetic and diabetic states. It was revealed that there was no association of calcium calculi with obesity (OR: 0.65, 95% CI: 0.29–1.45, *P* = 0.287), overweight (OR: 0.92, 95% CI: 0.48–1.76, *P* = 0.803), and HTN (OR: 1.07, 95% CI: 0.60–1.91, *P* = 0.823) in patients who had urinary stone disease. The presence of calcium calculi was significantly inversely associated with prediabetic (OR: 0.08, 95% CI: 0.04–0.17, *P* < 0.001) and diabetic states (OR: 0.21, 95% CI: 0.11–0.42, *P* < 0.001). The calcium calculi were further classified into calcium oxalate and calcium phosphate calculi for analysis. In patients with calcium oxalate calculi, there was a marked inverse association with prediabetic states (OR: 0.51, 95% CI: 0.30–0.87, *P* = 0.013). In contrast, there was a remarkable association with HTN (OR: 1.53, 95% CI: 1.03–2.28, *P* = 0.037) and a significant inverse association with prediabetic (OR: 0.46, 95% CI: 0.24–0.91, *P* = 0.025) and diabetic states (OR: 0.42, 95% CI: 0.25–0.70, *P* = 0.001) in patients who had calcium phosphate calculi. Furthermore, in those who had uric acid-predominant urinary stone disease, there was a remarkable association with prediabetic (OR: 20.11, 95% CI: 7.40–54.63, *P* < 0.001) and diabetic states (OR: 11.50, 95% CI: 4.41–29.97, *P* < 0.001). Additionally, it revealed that obesity was seemingly associated with uric acid calculi, but with no statistical significance (OR: 2.45, 95% CI: 0.91–6.62, *P* = 0.078). There was no obvious relationship between uric acid calculi and HTN (OR: 1.08, 95% CI: 0.54–2.17, *P* = 0.822). Besides, in patients who had urinary stone disease accompanied with urinary tract infection, there was a significant inverse association with the presence of calcium oxalate calculi (OR: 0.56, 95% CI: 0.39–0.80, *P* = 0.002). There was no association of uric acid calculi with concurrent urinary tract infection (OR: 0.93, 95% CI: 0.44–1.96, *P* = 0.841).

## 4. Discussions

Our main finding indicated that patients who had uric acid calculi were associated with prediabetic and diabetic states. The presence of uric acid calculi was not a risk factor of HTN or concurrent urinary tract infection in urolithiasis. To the best of our knowledge, this was the first investigation to elucidate the relationship of uric acid calculi to prediabetes and T2DM in the Taiwanese population.

Uric acid calculi are uncommon in the US, accounting for 7–10% of the urolithiasis subjected to analysis [17]. However, it was reported that the prevalence of uric acid calculi was higher in certain countries, including Israel (22–39.5%), Pakistan (28%), Germany (25%), Thailand (20%), and Japan (16%) [18–23], or in certain populations such as Americans of Asian descent (50%) [24]. Recently, there was emerging evidence indicating that uric acid calculi became more prevalent among patients with obesity, T2DM, and metabolic syndrome [11, 25]. This suggested that the pathogenetic formation of uric acid calculi could not merely be derived from abnormalities in uric acid metabolism but also be attributed to dietary habits and lifestyle [26, 27]. According to the literature, uric acid calculi were associated with obesity, metabolic syndrome, and T2DM [11–13, 28, 29]. It was consistent with our results in which urolithiasis with uric acid predominance was associated with prediabetic and diabetic states. Our results further indicated that uric acid calculi were also seemingly related to obesity, but there was not statistically significant. There was no association of uric acid calculi with HTN and concurrent urinary tract infection in patients undergoing surgical management for urolithiasis.

The pathogenetic mechanisms in the formation of uric acid urolithiasis were well-documented in principle. It was noticeable that urinary acidification, low urinary volume, and elevated uric acid concentration in the urine could contribute to the generation of uric acid calculi [23]. Of these, urinary acidification played the most crucial role in the formation of uric acid urolithiasis. It was also indicated that less renal ammonium excretion with low urinary pH, which could be linked with insulin resistance, was essential for the development in normouricosuric uric acid nephrolithiasis [29]. Further study demonstrated a strong inverse relationship between low urinary pH and peripheral insulin resistance, in which an increase of ammonium excretion in the urine was caused by hyperinsulinemia, thus leading to undue urinary acidity [30]. It was also proved that defective ammonia production was potentially linked with insulin resistance. In such case, increased free fatty acid in the peripheral blood was observed in insulin resistance and thus served as an alternative energy source for kidney cells, leading to less consumption of glutamine and therefore reduced ammonium production [23, 31, 32].

Our results revealed that both fasting glucose and HbA1c levels were significantly higher in the prediabetic and diabetic group in comparison with nondiabetic group. However, the median fasting glucose level was slightly higher than the referenced normal range. This could be explained by the acute physiological stress before undergoing surgical management for urolithiasis in our study population, thus leading to an increased glucose level. Similar phenomenon was also observed in the nonhypertensive group, in which the systolic blood pressure was slight higher than the referenced normal range. It was also interesting that our results indicated that calcium-predominant urolithiasis, particularly the calcium phosphate urolithiasis, was significantly inversely associated with prediabetic and diabetic states. According to the literature review, it was reported that diabetic patients had a significantly lower urinary calcium level in the 24-hour urine excretion than the nondiabetic individuals [33]. In contrast, another investigation indicated that significantly higher concentrations of calcium and phosphorus were observed in the 24-hour urine excretion of diabetic patients than that of the nondiabetic group [34]. The inverse association of calcium-predominant urolithiasis with prediabetic and diabetic states, however, could still not be fully explained. The most possible explanation of such phenomenon in our investigation was that there was certain selection bias in our study population, or the complementary change, putting in perspective that nearly 90% of the urolithiasis was calcium-predominant, and meanwhile, uric acid calculi which was disclosed to be associated with prediabetic and diabetic states as mentioned above accounted for 6.9% only.

It has been well documented that struvite stones, also known as magnesium ammonium phosphate, were invariably accompanied with urinary tract infection. However, the pathogenesis of urinary tract infection in other types of urolithiasis, such as calcium oxalate and calcium phosphate stones, has not been extensively surveyed. In a canine animal study, it was reported that infection-induced urolithiasis composed of struvite, calcium carbonate phosphate, ammonium acid urate, and calcium phosphate only as well as mixed stones with calcium oxalate and calcium phosphate was associated with positive results in the culture of urine or urocalculi [35]. In contrast, the urolithiasis is constituted of mixed stones with calcium oxalate and/or calcium phosphate, and uric acid and calcium phosphate were associated with both negative results of urine and urocalculi cultures [35], which was essentially compatible with our results. Nevertheless, recent studies indicated that there was an increased incidence of mixed stones composed of calcium oxalate and calcium phosphate cases in the culture-positive urolithiasis [36–38]. The relationship between urine stone composition and accompanied urinary tract infection in urolithiasis remains controversial and further research is required.

There were several limitations in our study. First of all, this investigation was the retrospective study design with small case number. Hence, the causal relationship between uric acid calculi and certain chronic disorders was indeterminate. Besides, it was reported that hyperuricemia and hyperlipidemia could be significantly associated with uric acid calculi, which was not assessed in this retrospective study because serum uric acid and lipid profiles were not preoperative routine examinations in patients undergoing surgical management for urolithiasis in our hospital. Thus, whether hyperuricemia and hyperlipidemia are confounding variables or not for the uric acid calculi remains uncertain. Also, patients who had uricosuric medications use, myeloproliferative neoplasms, congenital disorders involving uric acid metabolisms, or other risk factors of developing uric acid urolithiasis were not surveyed and thus the role of these risk factors remains to be determined in this study. Furthermore, only the relationship of calcium- and uric acid-predominant calculi with certain chronic disorders was analyzed due to limited number of cases of other types of urolithiasis. A multicenter full and matched cohort research for assessing the relationship of uric acid calculi to certain chronic diseases could be considered.

## 5. Conclusion

In conclusion, our study indicated that there was a remarkable association of uric acid calculi with prediabetic and diabetic states. Our results further showed that uric acid calculi were also seemingly associated with obesity, but there was no statistical significance. There was no obvious relationship of uric acid calculi with HTN and concurrent urinary tract infection in patients with urolithiasis undergoing surgical management.

## Figures and Tables

**Table 1:** Baseline characteristics in patients who underwent surgical management for urolithiasis.

Variables	Values	*P* value
Age (year)	55 (46–63)	
Gender (male/female)	462/204	
BMI (kg/m^2^)		
≧30	112 (16.8%)	
30 > BMI ≧ 24	348 (52.3%)	
<24	206 (30.9%)	
Underlying disorders		
Impaired fasting glucose	69 (10.4%)	
Type 2 diabetes mellitus	149 (22.4%)	
Hypertension	259 (38.9%)	
Concurrent UTI	184 (27.6%)	
Serum creatinine (mg/dL)	0.97 (0.75–1.13)	
Plasma glucose (mg/dL)		*P* < 0.001
Diabetic and prediabetic	138 (114–172)	
Nondiabetic	102 (92–118)	
HbA1c (%)		*P* < 0.001
Diabetic and prediabetic	6.8 (6.4–7.8)	
Nondiabetic	5.7 (5.4–5.9)	
Systolic blood pressure (mmHg)		*P* < 0.001
Hypertensive	146 (133–158)	
Nonhypertensive	131 (121–141)	
Diastolic blood pressure (mmHg)		*P* < 0.001
Hypertensive	85 (75–95)	
Nonhypertensive	80 (72–88)	
Predominant composition of urolithiasis		
Calcium oxalate	423 (63.5%)	
Calcium phosphate	174 (26.1%)	
Uric acid	46 (6.9%)	
Magnesium ammonium phosphate	11 (1.7%)	
Cystine	1 (0.2%)	
Other protein calculi	2 (0.3%)	
Mixed type	9 (1.4%)	

Data were presented as median (interquartile range, IQR) or number (percentage). BMI, body mass index; UTI, urinary tract infection.

**Table 2:** Multivariate analysis of urolithiasis composition and overweight, obesity, prediabetic and diabetic states, hypertension, and concurrent urinary tract infection.

	Calcium stone		Calcium oxalate		Calcium phosphate		Uric acid	
	OR (95% CI)	*P* value	OR (95% CI)	*P* value	OR (95% CI)	*P* value	OR (95% CI)	*P* value
BMI (kg/m^2^)								
BMI ≧ 30 versus BMI < 24	0.65 (0.29–1.45)	*P* = 0.287	1.07 (0.64–1.77)	*P* = 0.805	0.76 (0.43–1.32)	*P* = 0.327	2.45 (0.91–6.62)	*P* = 0.078
30 > BMI ≧ 24 versus BMI < 24	0.92 (0.48–1.76)	*P* = 0.803	1.20 (0.83–1.73)	*P* = 0.338	0.79 (0.53–1.18)	*P* = 0.257	1.50 (0.64–3.51)	*P* = 0.352
BMI ≧ 24 versus BMI < 24	0.90 (0.49–1.65)	*P* = 0.736	1.19 (0.84–1.69)	*P* = 0.338	0.78 (0.53–1.15)	*P* = 0.214	1.62 (0.72–3.64)	*P* = 0.243
Prediabetic and diabetic states								
Diabetic versus absent	0.21 (0.11–0.42)	*P* < 0.001	1.08 (0.70–1.64)	*P* = 0.739	0.42 (0.25–0.70)	*P* = 0.001	11.50 (4.41–29.97)	*P* < 0.001
Prediabetic versus absent	0.08 (0.04–0.17)	*P* < 0.001	0.51 (0.30–0.87)	*P* = 0.013	0.46 (0.24–0.91)	*P* = 0.025	20.11 (7.40–54.63)	*P* < 0.001
Diabetic and prediabetic versus absent	0.15 (0.08–0.27)	*P* < 0.001	0.83 (0.58–1.19)	*P* = 0.302	0.43 (0.28–0.67)	*P* < 0.001	14.58 (5.89–36.07)	*P* < 0.001
Hypertension								
Present versus absent	1.07 (0.60–1.91)	*P* = 0.823	0.73 (0.51–1.04)	*P* = 0.083	1.53 (1.03–2.28)	*P* = 0.037	1.08 (0.54–2.17)	*P* = 0.822
Concurrent UTI								
Present versus absent	0.47 (0.26–0.82)	*P* = 0.009	0.56 (0.39–0.80)	*P* = 0.002	1.41 (0.95–2.09)	*P* = 0.090	0.93 (0.44–1.96)	*P* = 0.841

Sex, age, BMI, prediabetic and diabetic states, HTN, and concurrent UTI were adjusted for multivariate regression analysis. OR, odds ratio; CI, confidence interval; BMI, body mass index; UTI, urinary tract infection.

## References

[1] Sorokin I., Mamoulakis C., Miyazawa K., Rodgers A., Talati J., Lotan Y. (2017). Epidemiology of stone disease across the world. *World Journal of Urology*.

[2] Wang W., Fan J., Huang G. (2017). Prevalence of kidney stones in mainland China: A systematic review. *Scientific Reports*.

[3] Wild S., Roglic G., Green A., Sicree R., King H. (2004). Global prevalence of diabetes: estimates for the year 2000 and projections for 2030. *Diabetes Care*.

[4] Pan W.-H., Yeh W.-T., Hwu C.-M., Ho L.-T. (2001). Undiagnosed diabetes mellitus in taiwanese subjects with impaired fasting glycemia: Impact of female sex, central obesity, and short stature. *Chinese Journal of Physiology*.

[5] Taylor E. N., Stampfer M. J., Curhan G. C. (2005). Diabetes mellitus and the risk of nephrolithiasis. *Kidney International*.

[6] Zeng G., Mai Z., Xia S. (2017). Prevalence of kidney stones in China: An ultrasonography based cross-sectional study. *BJU International*.

[7] Akarken I., Tarhan H., Ekin R. G. (2015). Visceral obesity: A new risk factor for stone disease. *Canadian Urological Association Journal*.

[8] Masterson J. H., Woo J. R., Chang D. C. (2015). Dyslipidemia is associated with an increased risk of nephrolithiasis. *Urolithiasis*.

[9] Obligado S. H., Goldfarb D. S. (2008). The association of nephrolithiasis with hypertension and obesity: A review. *American Journal of Hypertension*.

[10] Cupisti A., D’Alessandro C., Samoni S., Meola M., Egidi M. F. (2014). Nephrolithiasis and hypertension: possible links and clinical implications. *Journal of Nephrology*.

[11] Nerli R., Jali M., Guntaka A. K., Patne P., Patil S., Hiremath M. B. (2015). Type 2 diabetes mellitus and renal stones. *Advanced Biomedical Research*.

[12] Cameron M. A., Maalouf N. M., Adams-Huet B., Moe O. W., Sakhaee K. (2006). Urine composition in type 2 diabetes: Predisposition to uric acid nephrolithiasis. *Journal of the American Society of Nephrology*.

[13] Daudon M., Traxer O., Conort P., Lacour B., Jungers P. (2006). Type 2 diabetes increases the risk for uric acid stones. *Journal of the American Society of Nephrology*.

[14] Trinchieri A., Croppi E., Montanari E. (2017). Obesity and urolithiasis: evidence of regional influences. *Urolithiasis*.

[15] American Diabetes Association (2013). Diagnosis and classification of diabetes mellitus. *Diabetes Care*.

[16] The International Expert Committee (2009). International expert committee report on the role of the A1C assay in the diagnosis of diabetes. *Diabetes Care*.

[17] Ngo T. C., Assimos D. G. (2007). Uric Acid nephrolithiasis: recent progress and future directions. *Reviews in Urology*.

[18] Herbstein F. H., Kleeberg J., Shalitin Y., Wartski E., Wielinski S. (1974). Chemical and X ray diffraction analysis of urinary stones in Israel. *Israel Journal of Medical Sciences*.

[19] Rafique M., Bhutta R. A., Rauf A., Chaudhry I. A. (2000). Chemical composition of upper renal tract calculi in Multan. *Journal of the Pakistan Medical Association*.

[20] Hossain R. Z., Ogawa Y., Hokama S., Morozumi M., Hatano T. (2003). Urolithiasis in Okinawa, Japan: A relatively high prevalence of uric acid stones. *International Journal of Urology*.

[21] Prasongwatana V., Sriboonlue P., Suntarapa S. (1983). Urinary Stone Composition in North‐East Thailand. *British Journal of Urology*.

[22] Sakhaee K. (2014). Epidemiology and clinical pathophysiology of uric acid kidney stones. *Journal of Nephrology*.

[23] Maalouf N. M., Cameron M. A., Moe O. W., Sakhaee K. (2004). Novel insights into the pathogenesis of uric acid nephrolithiasis. *Current Opinion in Nephrology and Hypertension*.

[24] Portis A. J., Hermans K., Culhane-Pera K. A., Curhan G. C. (2004). Stone disease in the Hmong of Minnesota: Initial description of a high-risk population. *Journal of Endourology*.

[25] Maalouf N. M. (2011). Metabolic syndrome and the genesis of uric acid stones. *Journal of Renal Nutrition*.

[26] Caramia G., Di Gregorio L., Tarantino M. L., Galuffo A., Iacolino R., Caramia M. (2004). Uric acid, phosphate and oxalate stones: Treatment and prophylaxis. *Urologia Internationalis*.

[27] Ferraro P. M., Gambaro G. (2015). Uric acid nephrolithiasis. *Giornale Italiano Di Nefrologia*.

[28] Pak C. Y. C., Sakhaee K., Peterson R. D., Poindexter J. R., Frawley W. H. (2001). Biochemical profile of idiopathic uric acid nephrolithiasis. *Kidney International*.

[29] Sakhaee K., Adams-Huet B., Moe O. W., Pak C. Y. C. (2002). Pathophysiologic basis for normouricosuric uric acid nephrolithiasis. *Kidney International*.

[30] Abate N., Chandalia M., Cabo-Chan A. V., Moe O. W., Sakhaee K. (2004). The metabolic syndrome and uric acid nephrolithiasis: novel features of renal manifestation of insulin resistance. *Kidney International*.

[31] Vinay P., Lemieux G., Cartier P., Ahmad M. (1976). Effect of fatty acids on renal ammoniagenesis in in vivo and in vitro studies. *American Journal of Physiology*.

[32] Lemieux G., Vinay P., Gougoux A., Baverel G., Cartier P. (1977). Relationship between the renal metabolism of glutamine, fatty acids and ketone bodies. *Current problems in clinical biochemistry*.

[33] Zhu W., Mai Z., Qin J. (2016). Difference in 24-hour urine composition between diabetic and non-diabetic adults without nephrolithiasis. *PLoS ONE*.

[34] Devasia D., Meiyappan K., Mohanraj P. S., Narayanan D. L., Senthilkumar G. P., Yasir M. (2017). Association between adiponectin and insulin resistance in diabetic urolithiasis. *Oman Medical Journal*.

[35] Gatoria I. S., Saini N. S., Rai T. S., Dwivedi P. N. (2006). Comparison of three techniques for the diagnosis of urinary tract infections in dogs with urolithiasis. *Journal of Small Animal Practice*.

[36] Schwaderer A. L., Wolfe A. J. (2017). The association between bacteria and urinary stones. *Annals of Translational Medicine*.

[37] Tavichakorntrakool R., Prasongwattana V., Sungkeeree S. (2012). Extensive characterizations of bacteria isolated from catheterized urine and stone matrices in patients with nephrolithiasis. *Nephrology Dialysis Transplantation*.

[38] Golechha S., Solanki A. (2001). Bacteriology and chemical composition of renal calculi accompanying urinary tract infection. *Indian Journal of Urology*.

